# Carbolic Acid Applied to Diseased Lungs

**Published:** 1874-12

**Authors:** 


Carbolic Acid Applied to Diseased Lungs. — Prof. Mosler, of Germany, is now successfully treating phthisis, or pulmonary consumption, by making an incision through the wall of the chest, and drawing off the pus with a syringe, and afterwards washing out the ulcers with weak carbolic acid. No difficulty appears to have been experienced in the operation, and the condition of the patient was improved, the cough becoming less troublesome, and the febrile symptoms apparently moderated. One point, at least, is regarded as settled—and it is certainly one of great importance—so far as could be by a few experiments of this character, namely, that the local treatment of pulmonary cavities is undoubtedly practicable, and that the lung is really more tolerant of external interference than has been generally believed. The uses of carbolic acid are rapidly extending, and it bids fair to become one of the most valuable articles of the materia medica. It appears to be speedy death to diseased germs and fungus growths.

